# Pterostilbene Reduces Liver Steatosis and Modifies Hepatic Fatty Acid Profile in Obese Rats

**DOI:** 10.3390/nu11050961

**Published:** 2019-04-26

**Authors:** Leixuri Aguirre, Sara Palacios-Ortega, Alfredo Fernández-Quintela, Elizabeth Hijona, Luis Bujanda, María P. Portillo

**Affiliations:** 1 Nutrition and Obesity Group, Department of Nutrition and Food Sciences, Faculty of Pharmacy, University of the Basque Country (UPV/EHU) 01006 Vitoria, Spain; leixuri.aguirre@ehu.eus (L.A.); mariapuy.portillo@ehu.eus (M.P.P.); 2 Lucio Lascaray Research Centre, 01006 Vitoria, Spain; 3 CIBER Physiopathology of Obesity and Nutrition (CIBERobn), Institute of Health Carlos III, 01006 Vitoria, Spain; 4 Doisy Research Center, Biochemistry and Molecular Biology Department, Saint Louis University, St. Louis, MO 63104, USA; sara.palaciosortega@health.slu.edu; 5 Department of Gastroenterology, University of the Basque Country (UPV/EHU), Donostia Hospital, 20014 San Sebastián, Spain; elizabeth.hijona@biodonostia.org (E.H.); luis.bujanda@osakidetza.eus (L.B.); 6 Biodonostia Institute, 20014 San Sebastián, Spain; 7 CIBER Hepatic and Digestive Pathologies (CIBERehd), Institute of Health Carlos III, 20014 San Sebastián, Spain

**Keywords:** pterostilbene, liver steatosis, triacylglycerol metabolism, Zucker rat, fatty acid profile

## Abstract

Excessive fat accumulation within the liver is known as “simple hepatic steatosis”, which is the most benign form of non-alcoholic fatty liver disease (NAFLD). The aim of the present study was to determine whether pterostilbene improves this hepatic alteration in Zucker (*fa*/*fa*) rats. Animals were distributed in two experimental groups (*n* = 10) and fed a standard laboratory diet. Rats in the pterostilbene group were given a dose of 30 mg/kg body weight/d for six weeks. After sacrifice, serum glucose, transaminase, and insulin concentrations were quantified and the liver triacylglycerol content and fatty acid profile was analyzed. Different pathways of triacylglycerol metabolism in liver were studied, including fatty acid synthesis and oxidation, triglyceride assembly, fatty acid uptake, and glucose uptake. With pterostilbene administration, a reduction in insulin concentrations (consequently in the Homeostatic Model Assessment for Insulin Resistance (HOMA-IR)) and hepatic triacylglycerol content were observed. No effects were observed in pterostilbene-treated rats in the activity of *de novo* lipogenesis enzymes. An improvement in the fatty acid profile was observed in pterostilbene-treated rats. In conclusion, pterostilbene is a useful molecule to reduce liver steatosis. Its delipidating effect is due, at least in part, to reduced fatty acid availability and triacylglycerol synthesis, as well as to an increased very low-density lipoprotein assembly and fatty acid oxidation.

## 1. Introduction

Excessive fat accumulation within liver is known as “simple hepatic steatosis”, which is the most benign form of non-alcoholic fatty liver disease (NAFLD). It is a major cause of chronic liver disease in western societies, and the burden is expected to grow with the increasing incidence of obesity and metabolic syndrome, which are closely associated with it [1,2]. In obesity and insulin resistance, the increased adipocyte mass and the low response to insulin lead to an increased hydrolysis of triacylglycerols, resulting in the enhanced release of free fatty acids to the plasma, which are available for hepatic uptake [3,4].

Several animal models have been developed to study fatty liver disease. One of the most commonly used is the *fa*/*fa* Zucker rat, a model of genetic obesity that exhibits a spontaneous mutation in the leptin receptor. This alteration decreases receptor affinity and changes the transduction signal, leading to the development of severe obesity. Many metabolic syndrome features, such as hyperphagia, hyperglycemia, hyperinsulinemia, hypercholesterolemia, adipocyte hypertrophy, and hyperplasia, and muscle atrophy are also observed in this model [5]. Moreover, the liver synthesizes an excess of triacylglycerols and oxidizes a small amount of fatty acids, which lead to a rapid onset of hepatic steatosis [6].

The current treatment of liver steatosis is based on dietary energy restriction and physical activity [7,8]. In recent years, a great deal of attention has been paid to bioactive molecules, present in foods and plants, which can represent complementary tools, such as phenolic compounds. One of the most studied molecules is resveratrol (trans-3,5,4′-trihydroxystilbene), a phytoalexin occurring naturally in grapes, berries, and peanuts [9,10]. Several studies have been carried out using resveratrol and different models of liver steatosis in mice and rats [11,12]. These studies have demonstrated that resveratrol is able to reduce the liver triacylglycerol content. Moreover, in some reported studies, amelioration of parameters related to the liver steatosis effect has also been found in human beings [13].

Although positive effects on liver steatosis have been shown after resveratrol administration, this molecule shows an important problem related to its low bioavailability. Due to its strong metabolism in the intestine and liver, concentrations of resveratrol in plasma and tissues are low after oral administration [14,15]. As a consequence, there is a great interest in other resveratrol-related molecules whose bioavailability is greater. This is the case with pterostilbene, a dimethyl ether derivative of resveratrol (parent compound). The substitution of hydroxy groups with methoxy groups increases the transport of the molecule into cells and reduces its metabolization in gut and liver [16].

In this scenario, the aim of the present study was to determine whether pterostilbene improves steatosis, by using, as an animal model, the genetically obese Zucker rat. The analysis of several potential mechanisms of action underlying this effect was also undertaken.

## 2. Materials and Methods

### 2.1. Animals, Diet, and Experimental Design

The experiment was conducted with 20 Zucker (*fa*/*fa*) rats at 5 weeks old, purchased from Charles Rivers Laboratories (Barcelona, Spain), in accordance with the institution’s guide for the care and use of laboratory animals (approval document reference CEEA14/018). The rats were individually housed in polypropylene cages and kept in an isolated room with a constantly regulated temperature (22 ± 2 °C) under a 12:12 h artificial light/dark cycle (light on at 21:00). After a 6-day adaptation period, the rats were randomly distributed in two experimental groups of ten animals each and fed a standard laboratory diet (Harlan Laboratories, Barcelona, Spain). Rats in the pterostilbene group were orally given this phenolic compound at a dose of 30 mg/kg body weight/d through an orogastric catheter for 6 weeks. Pterostilbene was diluted in 1 mL of ethanolic solution (20%). Rats from the control group (Control) received only the vehicle. All animals had free access to food and water. Food intake and body weight were measured daily. Pterostilbene (99.9% purity) was synthesized according to published procedures [17].

At the end of the experimental period, and after an 8 to 12 h fasting period, rats were sacrificed by cardiac exsanguination under isoflurane anaesthesia. Serum was obtained from blood samples after centrifugation (1000× *g* for 10 min, at 4 °C). Liver and adipose tissue from epididymal, perirenal, mesenteric, and subcutaneous anatomical locations were dissected, weighed, and immediately frozen in liquid nitrogen. All samples were stored at −80 °C until analysis.

### 2.2. Serum Parameters

Serum glucose, triacylglycerol, and transaminase concentrations were carried out by using Cobas 8000 analyzer (Roche, Basel, Switzerland). A serum non-esterified free fatty acids (NEFAs) (FUJIFILM Wako Diagnostics U.S.A., Mountain View, CA, USA) insulin concentration was measured by using the commercial ELISA kit (Roche Diagnostics GmbH, Mannheim, Germany, Millipore, Darmstadt, Germany).

The Homeostatic Model Assessment for Insulin Resistance (HOMA-IR) was calculated from basal insulin and glucose values using Matthews’ formula [18]:

HOMA-IR = [Fasting glucose (mmol/L) × fasting insulin (mU/L)]/22.5

### 2.3. Steatosis Assessment

Total lipids were extracted from the liver, following the method described by Folch [19]. The lipid extract was dissolved in isopropanol. Triacylglycerol content was measured by using a commercial kit (Spinreact, Barcelona, Spain).

Moreover, a histological study was performed. Just after killing, a piece of liver was placed in 10% buffered formalin and subsequently embedded in paraffin. Liver sections were stained with haematoxylin and eosin using standard techniques. Two sections per sample (with two images per section) were viewed without knowing the treatment group to which each animal belonged. Biopsies were classified into four grades depending on fat accumulation using the Brunt et al. classification, assigning grade 0 when no fat was found in the liver, grade 1 when fat vacuoles were seen in less than 33% of hepatocytes, grade 2 when 33–66% of hepatocytes were affected by fat vacuoles, and grade 3 when fat vacuoles were found in more than 66% of hepatocytes [20]. Two experienced pathologists who were masked to the experiment evaluated all samples and reached an agreement.

### 2.4. Enzyme Activities

To determine the fatty acid synthase (FAS) activity, 500 mg of liver were homogenized in 5 mL of a buffer (pH 7.6) containing 150 mM KCl, 1 mM MgCl_2_, 0.5 mM dithiothreitol, and 10 mM *N*-acetyl-cysteine. Enzyme isolation was performed by centrifugation at 100,000× *g*, 4 °C for 40 min. The supernatants obtained were stored at −80 °C for further analysis. FAS activity was monitored spectrophotometrically by measuring the NADPH consumption, as previously described [21,22], and the results were expressed as nanomoles NADPH consumed per minute, per mg of protein.

The activity of carnitine palmitoyltransferase-1a (CPT-1a) was assessed in the mitochondrial/peroxisomal fraction. Liver samples (500 mg) were homogenized in 1.5 mL of a buffer (pH 7.4) containing 25 mM sucrose, 1 mM EDTA, and 10 mM Tris-HCl. After centrifugation at 700× *g*, 4 °C for 10 min, the supernatant fraction was again centrifuged at 12,000× *g*, 4 °C for 15 min. Pellets were resuspended in 500 μL of a buffer (pH 7.4) composed of 70 mM sucrose, 220 mM mannitol, 2 mM HEPES, and 1 mM EDTA. CPT-1a activity was assayed by using the Bieber method [23], based on the amount of coenzyme A released from palmitoyl-CoA in the presence of 5,5′-dithiobis-(2-nitrobenzoic acid) (DTNB). The pellet protein content was determined by the Bradford method [24]. CPT-1a activity was expressed as nmol CoA, formed per minute per mg protein.

To determine the citrate synthase (CS) activity, 200 mg of liver were homogenized in 5 mL of 0.1 M Triethanolamine-HCl buffer (pH 8.0) and diluted tenfold in the same buffer. Homogenates were incubated for 5 min at 30 °C with 0.1 M Tris-HCl of a buffer containing 0.1 mM DTNB, 0.25% Triton X-100, 0.5 mM oxalacetate, and 0.31 mM acetyl-CoA, and readings were taken at 412 nm. CS activity was expressed as nanomoles CoA formed per minute, per mg of protein [25].

To assess the assembly of very low-density lipoproteins by the liver, microsomal triglyceride transfer protein (MTP) activity was determined fluorimetrically by using a commercial kit (Sigma-Aldrich, St. Louis, MO, USA). MTP activity was expressed as a percentage of triacylglycerol transfer/mg protein/hour [26].

### 2.5. Liver RNA Extraction and Real Time RT-PCR

The total RNA from 100 mg of liver samples was isolated using TRIzol^®^ reagent (Invitrogen, Carlsbad, CA, USA), according to the manufacturer’s instructions. RNA concentration and quality were determined using a Nanodrop spectrophotometer (Thermo Scientific, Wilmington, DE, USA). Each RNA sample was subsequently submitted to two DNase treatments (Ambion, Applied Biosystems, Austin, TX, USA) to ensure the complete removal of possible contaminating genomic DNA. Next, 1.5 μg of purified total RNA was used for the synthesis of first-strand cDNA, using iScript reverse transcriptase (BioRad, Hercules, CA, USA), following the protocol provided by the company.

SYBR-Green real-time reverse transcription-polymerase chain reaction technology (Applied Biosystems, Foster City, CA, USA) was used to perform the relative quantification of the fatty acid translocase/scavenger receptor cluster of differentiation (CD36) and respiratory electron transport chain complex II (cox-2) mRNA expression. β-actin was selected as the invariant internal control for reverse transcription quantitative polymerase chain reaction (RT-qPCR) and subsequent normalization. Specific primers were synthesized commercially (Eurogentec, Seraing, Belgium; Integrated DNA Technologies, Leuven, Belgium). Primer sequences are detailed in Table 1. The complementary DNA (cDNA) was amplified in an iCycler™–MyiQ™ Real-Time PCR Detection System (Bio-Rad, Hercules, CA, USA), according to the standard conditions. The SYBR-Green real-time reverse transcription-polymerase chain reaction parameters were as follows: Initially 2 min at 50 °C, denaturation at 95 °C for 10 min, followed by 40 cycles of denaturation at 95 °C for 15 s, and combined annealing and extension for 1 min. Annealing was performed at 61.5 °C for CD36 and 60 °C for cox-2 and β-actin. The relative quantification of gene expression was calculated by the 2^−ΔΔCt^ method [27]. Data from pterostilbene-treated groups were referenced to the control group.

### 2.6. Liver Total Protein Extraction and Western Blot

The total number of proteins from 100 mg of liver samples were extracted using ice-cold PBS (0.15 M NaCl, 3 mM KCl, 3 mM NaH_2_PO_4,_ and 7.5 mM Na_2_HPO_4_; pH 7.4) containing 1 mM PMSF and 0.1 mM iodoacetamide. Proteins were purified from the lysate by centrifugation at 800× *g*, 4 °C for 5 min and were stored at −80 °C for further analysis.

Nuclear proteins were extracted for sterol regulatory element-binding protein 1 (SREBP-1c) and peroxisome proliferator-activated receptor α (PPARα) quantification, performing a centrifugation at 300× *g*, 4 °C for 10 min after ice-cold PBS with inhibitors homogenization. P-40 reagent (10% *v*/*v*) was added to each protein sample before carrying out another centrifugation at 14,000× *g*, 4 °C for 1 min. Pellets were resuspended in 100 μL of a specific buffer, composed of 10 mM HEPES, 0.1 mM EDTA, 1.5 mM MgCl_2_ 6H_2_O, 420 mM NaCl, glycerol 10% *v*/*v*, 5 mM DTT, 0.5 mM phenylmethylsulfonyl fluoride (PMSF), and 0.05 mM iodoacetamide and were subsequently incubated at 4 °C under rotation during 30 min, shaking them every 15 min.

Supernatants were obtained after centrifugation at 14,000× *g*, 4 °C for 10 min and were stored at −80 °C for further analysis. After subjecting all the samples to the Western blot analysis, total protein content was quantified by the Bradford method. The number of proteins for each desirable target were denatured by heat, fractionated by sodium dodecyl sulfate - polyacrylamide gel electrophoresis (SDS-PAGE), and transferred to polyvinyl difluoride membranes (Millipore, Bradford, MA, USA), which had been previously activated by a methanol treatment. Next, membranes were blocked with 5% bovine serum albumin (BSA) in 0.01% tris-buffered saline with 0.1% Tween 20 (TBST) for 2 h, before being incubated overnight with the suitable primary antibody dilution at 4 °C. After washing the membranes three times with TBST, they were incubated with an appropriate dilution of horseradish peroxidase (HRP)-conjugated secondary antibody for another 2 h. Finally, after three more washes, specific immunoreactive bands were detected by a chemiluminescent ECL assay kit (Thermo Scientific, USA). The intensity of the bands was determined by densitometric analysis and quantified by a ChemiDoc MP imaging system (Bio-Rad, USA). Primary antibodies for acetyl-CoA carboxylase (ACC) (10 μg of protein, 1:1000 antibody dilution), phosphorylated ACC (pACC; 10 μg of protein, 1:1000 antibody dilution) from Cell Signaling Technology (Beverly, MA, USA), and SREBP-1c (15 μg of protein, 1:1000 antibody dilution), PPARα (30 μg of protein, 1:1000 antibody dilution), diacylglycerol O-acyltransferase 2 (DGAT2) (100 μg of protein, 1:500 antibody dilution), mitochondrial transcription factor A (mtTFA) (100 μg of protein, 1:500 antibody dilution), and mitochondrial uncoupling protein 2 (UCP2) monomer and dimer (100 μg of protein, 1:500 antibody dilution) were purchased from Santa Cruz Biotechnology (Santa Cruz, CA, USA). A specific secondary HRP-labeled rabbit (1:1000 antibody dilution), mouse (1:1000 antibody dilution), or goat (1:1000 antibody dilution) antibodies were employed depending on the primary antibody origin. β-actin (1:1000 antibody dilution) was used as an invariant internal control for sample normalization.

### 2.7. Fatty Acid Profile of Hepatic Triacylglycerols and Phospholipids

The analysis of the fatty acid composition of hepatic triacylglycerols and phospholipids was performed following a previously described procedure (Arias et al., 2014). Frozen samples (0.3 g) of liver were weighed, kept at 0 °C in an ice bath, and homogenized in chloroform:methanol 2:1 (*v*/*v*) [19]. After drying under nitrogen, the sample was dissolved in 1 mL of chloroform and the lipid species were separated by using Sep-Pack Vac 3cc (500 mg) amino propyl (NH_2_) cartridges (SPE columns) (Waters, Catalog number WAT02084). The correct separation procedure was confirmed by thin layer chromatography.

Sodium methoxide in methanol (0.5 M) (Sigma–Aldrich, Steinheim, Germany) was added (500 µL) to lipid species extracts to prepare fatty acid methyl esters (FAMEs). FAMEs were solved in 500 µL of hexane containing 50 ppm of butylated hydroxytoluene as a stabilizer and were transferred to gas chromatography (GC) vials and analyzed with a Hewlett Packard HP6890 gas chromatograph (Hewlett Packard, Palo Alto, CA, USA), connected to an automatic injector (Hewlett Packard HP7673), and equipped with a flame ionization detector (FID). Samples were continuously maintained at 4 °C with a refrigerated recirculation bath (Frigiterm TFT-10, JP Selecta, Barcelona, Spain). The analytical column was a fused silica capillary SP-2380 column (100 m; 0.25 mm i.d.; 0.20 µm film thickness), purchased from Supelco (Bellafonte, PA, USA). The oven temperature was initially programmed at 150 °C (hold 4 min) and raised to 220 °C at a 4 °C/min rate (hold 25 min). Injection (1 µL) was run in split (40:1) mode. Helium was the carrier gas at constant flow (1 mL/min) and make-up gas for the FID. The injector was maintained at 200 °C and the detector at 250 °C. FAMEs were identified by comparison with retention times with standards (Sigma, St. Louis, MO, USA), and they were expressed as a percentage of total fatty acids in each lipid species (triacylglycerols or phospholipids).

### 2.8. Statistical Analysis

Results were presented as mean ± standard error of the mean (SEM). Statistical analysis was performed using IBM SPSS Statistics 24.0. All the parameters were normally distributed, according to the Shapiro–Wilk’s test. The Student’s *t* test was used for comparisons between both experimental groups. Significance was assessed at the *p* value < 0.05 level. Effect size was calculated according to Cohen’s *d* formula with the recommendation of 0.2, 0.5, and 0.8 for small, moderate, and large effects, respectively.

## 3. Results

### 3.1. Liver Weight and Hepatic Steatosis

**Figure 1:**
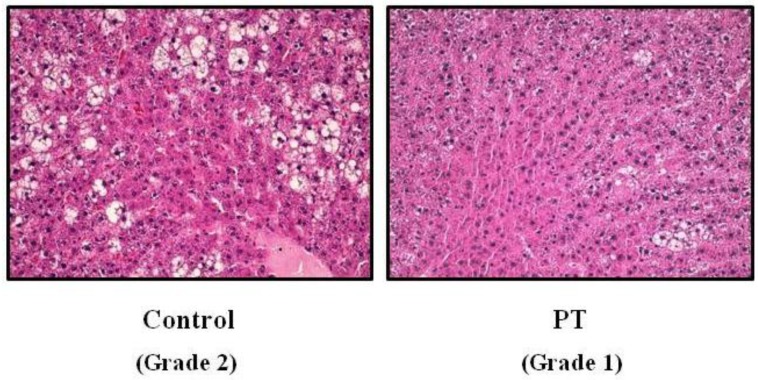
Histological study in liver from *fa*/*fa* Zucker rats either treated or not with pterostilbene (30 mg/kg body weight/day) for six weeks. Hematoxylin and eosin staining of liver tissue × 40.

Pterostilbene significantly reduced adipose tissue mass (*p* = 0.04). Interestingly, a lower liver weight was observed in rats treated with pterostilbene. Although the differences were statistically non-significant (*p* = 0.092), the effect size was rated as moderate (Cohen’s *d* = −0.78). Triacylglycerol content was also reduced by pterostilbene (−16.9%). When steatosis was evaluated by microscopy, fat infiltration that reached grade 2 in the control rats was reduced to grade 1 in the pterostilbene-treated group (Figure 1). Moreover, the percentage of samples showing grade 3 steatosis were reduced from 15.5% in the control rats to 3.1% in pterostilbene-treated animals. No differences were observed in food intake between both experimental groups (Table 2).

As far as serum biochemical measurements are concerned, the administration of pterostilbene induced a significant reduction in glucose (*p* = 0.03) and insulin concentrations (*p* = 0.03). Consequently, HOMA-IR was also significantly reduced (*p* = 0.02) (Table 2). No significant differences were observed between both experimental groups in triacylglycerol, NEFAs, and serum transaminase concentrations (Table 2).

### 3.2. Enzyme Activities

**Figure 2:**
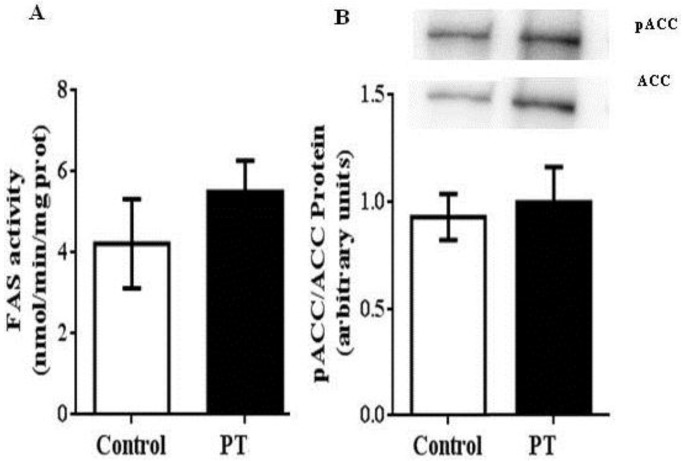
Fatty acid synthase (FAS) (**A**) and acetyl CoA-carboxylase (ACC) (**B**) activities in liver from *fa*/*fa* Zucker rats either treated or not with pterostilbene (30 mg/kg body weight/day) for six weeks. Values are means ± SEM (*n* = 10). Differences between groups were determined by the Student’s *t* test. Statistical significance was set at the *p* < 0.05 level.

Regarding *de novo* lipogenesis, in the present study the activity of FAS was measured by spectrophotometry. In addition, the ratio phosphorylated ACC/total ACC, measured by Western blot, was used as an index of ACC activity because this enzyme is unactivated by phosphorylation. In both cases, no effects were observed in pterostilbene-treated rats (Figure 2A,B).

**Figure 3:**
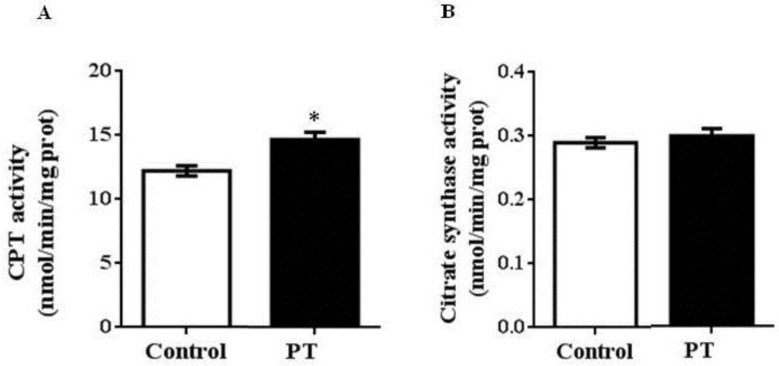
Carnitine palmitoyltransferase-1a (CPT-1a) (**A**) and citrate synthase (CS) (**B**) activities in liver from *fa*/*fa* Zucker rats either treated or not with pterostilbene (30 mg/kg body weight/day) for 6 weeks. Values are means ± standard error of the mean (SEM) (*n* = 10). Differences between groups were determined by the Student’s *t* test. Statistical significance was set at the *p* < 0.05 level. * *p* < 0.05.

We also measured the activities of two enzymes involved in fatty acid oxidation. In this case, a significant increase was observed in CPT-1a activity in PT group when compared to the control group (*p* = 0.003) (Figure 3A), whereas no differences were found in CS activity between both experimental groups (Figure 3B).

**Figure 4:**
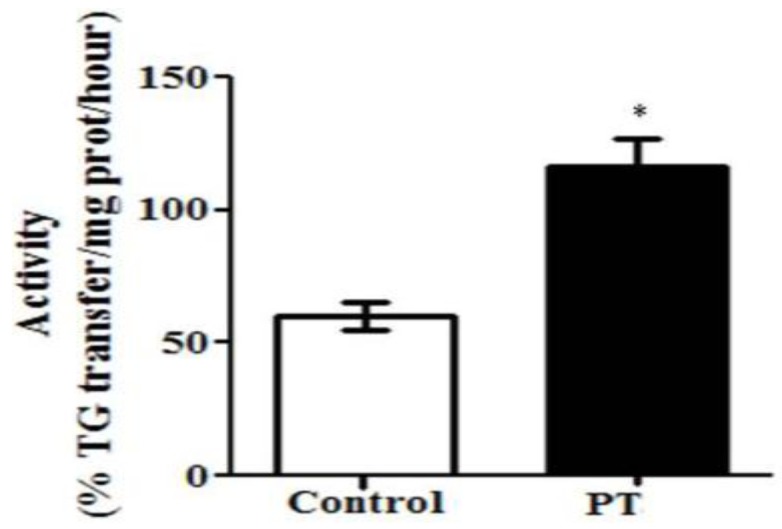
Microsomal triglyceride transfer protein (MTP) activity in liver from *fa*/*fa* Zucker rats either treated or not with pterostilbene (30 mg/kg body weight/day) for six weeks. Values are means ± SEM (*n* = 10). Differences between groups were determined by the Student’s *t* test. Statistical significance was set at the *p* < 0.05 level. * *p* < 0.05.

With regard to MTP, a significant increase was observed in the PT group (*p* = 0.001) (Figure 4).

### 3.3. Gene Expression

**Figure 5:**
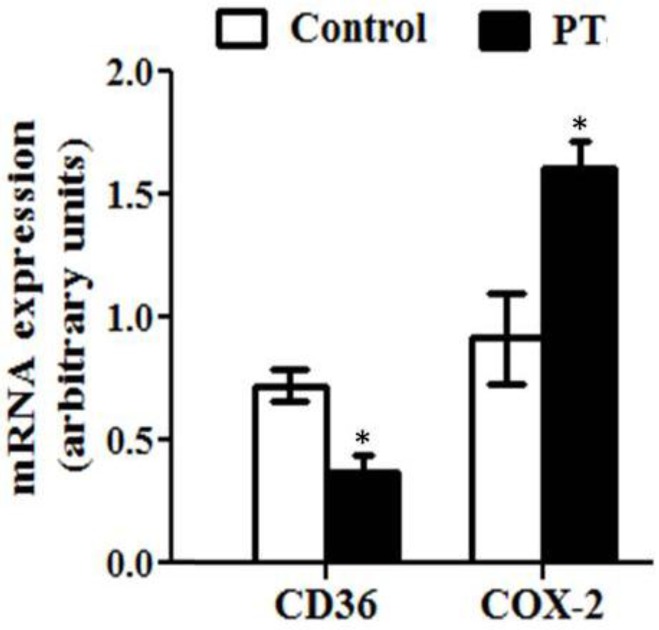
Fatty acid translocase/scavenger receptor cluster of differentiation (CD36) and respiratory electron transport chain complex II (cox-2) gene expression in liver from *fa*/*fa* Zucker rats either treated or not with pterostilbene (30 mg/kg body weight/day) for six weeks. Values are means ± SEM (*n* = 10). Differences between groups were determined by the Student’s *t* test. Statistical significance was set at the *p* < 0.05 level. * *p* < 0.05.

The expression of several genes involved in lipid metabolism was also assessed in the present study. The expression of CD36, a fatty acid transporter, was significantly decreased by pterostilbene (*p* = 0.01) (Figure 5). Moreover, the mRNA level of cox-2, a mitochondrion-encoded protein located in the inner mitochondrial membrane, was significantly increased in rats from the PT group (*p* = 0.01) (Figure 5).

### 3.4. Protein Expression

**Figure 6:**
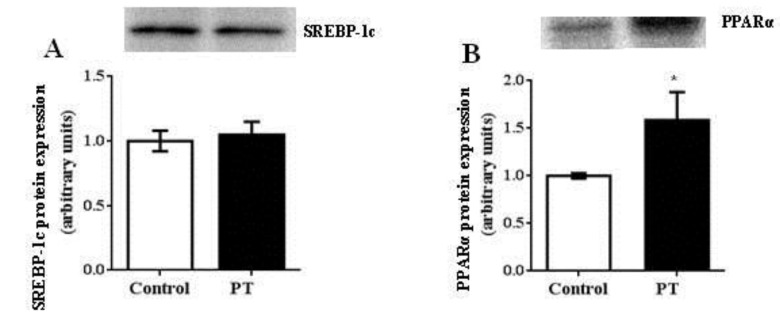

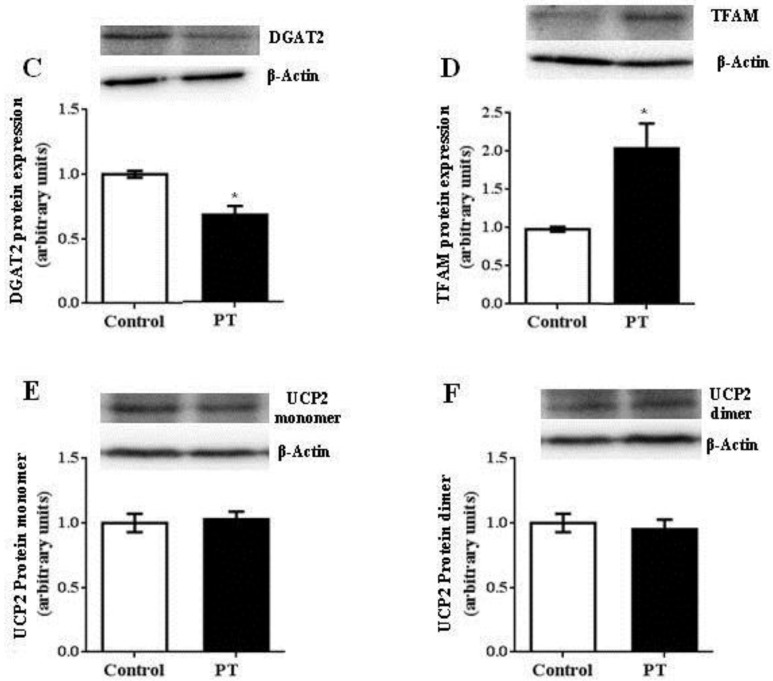
Sterol regulatory element-binding protein-1 (SREBP-1c) (**A**), peroxisome proliferator-activated receptor α (PPARα) (**B**), diacylglycerol O-acyltransferase 2 (DGAT2) (**C**), mitochondrial transcription factor A (mtTFA or TFAM) (**D**) and uncoupling protein 2 (UCP2) (**E**,**F**) in liver from fa/fa Zucker rats either treated or not with pterostilbene (30 mg/kg body weight/day) for six weeks. Differences between groups were determined by the Student’s *t* test. Statistical significance was set at the *p* < 0.05 level. * *p* < 0.05.

Pterostilbene administration did not modify protein expression of SREBP-1c, the transcription factor that regulates the expression of lipogenic enzymes (ACC and FAS) (Figure 6A). In contrast, protein expression of PPARα, which plays a key role in the transcription of genes involved in fatty acid oxidation, was up-regulated by pterostilbene treatment (*p* = 0.04) (Figure 6B). In addition, mtTFA, involved in mitochondria biogenesis, was also significantly increased in the PT group when compared to the control group (*p* = 0.01) (Figure 6C). Finally, protein expression of DGAT2, an enzyme with a crucial role in the assembly of triacylglycerols, was significantly decreased in the PT group (*p* = 0.02) (Figure 6D) when compared to the control group, while the UCP2 expression remained unchanged, in both monomeric and dimeric forms (Figure 6E,F).

### 3.5. Fatty Acid Profile

The fatty acid profile of the hepatic triacylglycerols was slightly modified by the pterostilbene treatment (Table 3). Linoleic acid (C18:2 *n*-6) and linolenic acid (C18:3 *n*-3) were significantly increased in the liver from rats treated with pterostilbene. Accordingly, the sum of polyunsaturated fatty acids (PUFA) was also increased.

As regards to the fatty acid profile of hepatic phospholipids, the sum of polyunsaturated fatty acids (PUFA) was also statistically increased in pterostilbene-treated animals. This was mainly due to the increase in docosahexaenoic acid (C22:6 *n*-3), although other polyunsaturated fatty acids (linoleic acid and arachidonic acid, 20:4 *n*-6) were also increased without reaching statistical significance (Table 4). In addition, vaccenic acid (C18:1 *cis*-11) content was statistically reduced after the pterostilbene treatment. This decrease, along with that of palmitoleic acid (C16:1 *cis*-9) led to a reduction in total monounsaturated fatty acids (MUFA), which was nearly statistically significant (*p* = 0.06; Cohen’s *d* = −0.97).

## 4. Discussion

As indicated in the Introduction section, an important advantage of pterostilbene, with regard to its parent compound resveratrol, relies on its higher bioavailability. As a matter of fact, the presence of two methoxy groups, instead of two hydroxy groups in its chemical structure, makes it less susceptible to the intestinal and hepatic metabolism [16]. It is important to take in mind that the number and position of the hydroxy/methoxy groups in the stilbene-based molecular structure can have an influence on the beneficial actions on health [17,28]. However, this issue has been poorly studied so far and thus it needs further investigation. With regard to positive effects of pterostilbene on steatosis, no studies have been performed. In this scenario, taking into account that resveratrol has been reported to reduce liver fat accumulation, in the present study we analyzed the effects of pterostilbene on steatosis in obese Zucker rats, an animal model which shows fatty liver.

It is well known that the analysis of liver biopsy remains as the gold standard for fatty liver disease diagnosis. Consequently, in the present study, histological analysis of liver was carried out. Obese Zucker rats (control group) showed a moderate steatosis because this analysis showed grade 2, according to Brunt scale, in terms of fat infiltration. Pterostilbene partially reduced this alteration because rats from the PT group showed grade 1, meaning that the number of hepatocytes affected was reduced. This delipidating effect was also observed when the hepatic triacylglycerol content was quantified by using a spectrophotometric method; in fact, rats treated with the phenolic compound showed a reduction of 17%. This effect was not due to a reduction in energy intake because no differences were found between both experimental groups. These results show that the presence of two methoxy groups in the pterostilbene molecule allows this phenolic compound to maintain the delipidating effect on fatty liver showed by its parent compound resveratrol. Unexpectedly, no reductions were observed in transaminases in the PT group. Concerning this issue, it is important to emphasize that, although transaminases are commonly used as biomarkers of fatty liver, they are not reliable markers because they can be normal, even in advanced NAFLD [29,30]. In addition, there are data in the literature showing reductions in liver steatosis that were not accompanied by decreases in transaminase concentrations [31,32].

One of the main factors involved in the ethipatogenesis of liver steatosis is insulin resistance. Indeed, it is one of the multiple hits predisposing to the development of NAFLD [33]. This metabolic alteration results in increased fatty acid release from adipocytes, due to increased lipolysis. As a result, liver is submitted to an excessive flux of this lipid species that contributes to triacylglycerol accumulation. In the present study, rats treated with resveratrol showed decreased adipose tissue size and ameliorated insulin resistance, as shown by reduced values of HOMA-IR, suggesting reduced fatty acid release from adipose depots. This can be one of the mechanisms underlying the reduction in hepatic triacylglycerols induced by this phenolic compound. Nevertheless, a limitation of the study is that lipolysis was not experimentally assessed.

The amount of triacylglycerols accumulated in hepatocytes is regulated by various metabolic processes, fatty acid uptake, fatty acid synthesis, and esterification on the one hand (“input”) and fatty acid oxidation and triacylglycerol export on the other hand (“output”). Steatosis occurs when “input” exceeds “output” [34,35]. In order to analyze the mechanisms of action underlying the delipidating action of pterostilbene, we assessed its effects on several parameters related to these processes.

As far as fatty acid uptake is concerned, the CD36 gene expression was measured. The CD36 transporter is involved in the uptake of both diet and lipolysis-derived free fatty acids. It should be emphasized that approximately two-thirds of hepatic triacylglycerols are derived from this pathway [36]. In obese Zucker rats, the increased adipocyte mass, together with insulin resistance, leads to an increased release of free fatty acids, which, thus, are available for hepatic uptake; they enter the hepatocyte leading to the stimulation of hepatic triacylglycerol production. In the present study, pterostilbene-treated rats showed a significant reduction in CD36 expression. According to these results, taking into account the reduction in adipose tissue mass and insulin resistance, it can be proposed that the availability of fatty acids coming from blood was reduced in the liver of rats treated with pterostilbene when compared to the control rats.

Another source of fatty acids for triacylglycerol synthesis is *de novo* lipogenesis. Normally, this metabolic pathway accounts for 5% of hepatic fat content, but when obesity is developed, it can represent up to 25% [37]. In fact, it has been demonstrated that this process is highly increased in obese Zucker rats [6]. In the present study, protein expression of SREBP-1c, the transcription factor that controls the two main lipogenic enzymes, ACC and FAS, was measured, and no differences were found between both experimental groups. Similarly, the activity of these enzymes remained unchanged in the PT group. These results show that *de novo* lipogenesis was not affected by pterostilbene treatment. In a previous study from our group, we also observed this lack of effect when pterostilbene was administered to Wistar rats, together with a high-fat, high-sucrose diet [38]. This fact reveals a difference between pterostilbene and resveratrol, because a great number of studies have reported that resveratrol reduces liver fat, in part, by inhibiting hepatic *de novo* lipogenesis [37,39].

DGAT2, one of two enzymes which catalyze the final reaction in the synthesis of triacylglycerols, in which diacylglycerol is covalently bound to long-chain fatty acyl-CoAs, is overexpressed in NAFLD [40,41]. A significant reduction in its protein expression was observed in the PT group. This result shows that pterostilbene reduced liver capacity for triacylglycerol synthesis, not only by decreasing fatty acid disposal, but also by inhibiting triacylglycerol synthesis.

Liver fatty acid oxidation is reduced in obese Zucker rats [6]. When we analyzed the effects of pterostilbene on this process, we observed that rats treated with this phenolic compound showed an increased protein expression of PPARα, the transcription factor that controls fatty acid oxidation. In addition, the activity of CPT-1a, a rate-limiting enzyme in beta-oxidation, involved in the transport of fatty acids into the mitochondria matrix, was also increased. In order to know if pterostilbene increased the number of mitochondria in liver, we further analyzed the protein expression of mtTFA, a mitochondriogenesis marker, and gene expression of cox-2, a mitochondrion-encoded protein embedded in the lipid bilayer of the inner mitochondrial membrane, which is a critical component of the oxidative phosphorylation pathway. In both cases, greater values were found in pterostilbene-treated rats. These results indicate that mitochondrial biogenesis and fatty acid oxidation were positively modulated by pterostilbene. Fatty acids fated to oxidation are not available for triacylglycerol synthesis and thus can limit steatosis.

Finally, the activity of MTP was increased in liver from rats treated with pterostilbene. This enzyme transfers triacylglycerols to nascent apolipoprotein B, producing very low density lipoproteins (VLDL) and removing lipid from hepatocytes. Consequently, it can be stated that this effect contributed to the steatosis reduction induced by this phenolic compound. The increase in MTP activity was not accompanied by enhanced values of serum triacylglycerols, suggesting that other processes taking place in tissues different from liver, and also involved in serum VLDL balance, could be modified by pterostilbene. This would be the case of the uptake of fatty acids from triacylglycerols circulating as VLDL, mediated by lipoprotein-lipase in skeletal muscle and white adipose tissue. A limitation of this work is that the study of tissues other than liver were not included.

The role of UCP2 in NAFLD development has been intensively studied, but reported studies are controversial. Some studies have shown that hepatocellular UCP2 expression is increased in NAFLD, indicating its potential role in disease development [42,43,44,45]. However, other studies have demonstrated that UCP2 deficiency caused diminished hepatic utilization and fatty acid clearance and thus it can lead to liver steatosis [46]. Moreover, it has been reported that obesity-related fatty liver is unchanged in mice deficient in mitochondrial UCP2 [44]. Thus, in the present study we analyzed the UCP2 protein expression to gain more insight concerning this issue. Unfortunately, no changes were observed after pterostilbene treatment, meaning that, irrespective of the positive or negative effect of UCP2 on steatosis, the delipidating effect of this phenolic compound was not mediated by this uncoupling protein.

Finally, as regards to the lipid profile, hepatic steatosis is characterized by an altered profile with an increase in monounsaturated and a decrease in polyunsaturated fatty acids, mainly those of long -chain docosahexaenoic acid [47]. These modifications lead to alterations in signal transduction associated with PPARα and decreased membrane fluidity [48]. Unfortunately, we cannot compare our data with those from Zucker lean littermates to know the changes in the triacylglycerol and phospholipid fatty acid profile. Nevertheless, taking into account the increase observed in PUFAs, triacylglycerols, and that seen in the phospholipids, as well as the trend to decrease the MUFAs in this lipid species, reversion of alterations induced in fatty liver, and thus a positive effect on the fatty acid composition on the steatotic liver, can be attributable to pterostilbene.

In conclusion, pterostilbene arises as a useful molecule to reduce liver steatosis. Its delipidating effect is due, at least in part, to reduced fatty acid availability, triacylglycerol synthesis, as well as to an increased very low-density lipoprotein assembly and increased fatty acid oxidation.

## Figures and Tables

**Table 1:** Primers for PCR amplification of each studied gene.

	Sense Primer	Antisense Primer
CD36	5′-GGT GTG CTC AAC AGC CTT ATC-3′	5′-TTA TGG CAA CCT TGC TTA TG-3′
Cox-2	5′-AAC AAT TCT CCC AGC TGT CAT TC-3′	5′-AGT CAA AGC ATA GGT CTT CAT AGT C-3′
β-actin	5′-ACG AGG CCC AGA GCA AGA-3′	5′-GGT GTG GTG CCA GAT CTT CTC-3′

CD36: Cluster of differentiation 36; Cox-2: mitochondrial subunit 2 of cytochrome c oxidase II.

**Table 2:** General parameters from obese Zucker rats treated or not with pterostilbene (30 mg/kg body weight/day) for six weeks.

	Control	PT		Effect Size ^a^
**Initial body weight** (g)	203 ± 4	204 ± 6		
**Body weight** (g)	372 ± 10	347 ± 12	NS	
**Food intake** (g/day)	24.7 ± 1.8	22.6 ± 1.2	NS	
**Adipose tissue weight** (g)	49.4 ± 1.8	43.5 ± 1.9	*p* = 0.04	−1.01
**Liver weight** (g)	18.4 ± 0.5	16.9 ± 0.7	NS	
**Hepatic triacylglycerol** (mg/g)	60.0 ± 3.6	49.8 ± 6.9	NS	
**Serum glucose** (mg/dL)	139 ± 11	108 ± 5	*p* = 0.03	−1.16
**Serum insulin** (mU/L)	54.1 ± 15.1	19.0 ± 4.4	*p* = 0.03	−1.01
**HOMA-IR**	18.7 ± 4.8	4.5 ± 1.5	*p* = 0.02	−1.28
**Serum triacylglycerols** (mg/dL)	277.7 ± 36.2	274.8 ± 50.8	NS	
**Serum NEFAs** (mmol/L)	2.3 ± 0.4	2.1 ± 0.1	NS	
**Serum AST** (U/L)	154 ± 22	143 ± 17	NS	
**Serum ALT** (U/L)	102 ± 8	90 ± 7	NS	

Alanine transaminase (ALT); Aspartate transaminase (AST); homeostatic model assessment of insulin resistance (HOMA-IR); non-esterified free fatty acids (NEFAs); Pterostilbene (PT). ^a^ Cohen’s recommendation for the interpretation of effect size: Little effect (*d* ≥ 0.20); moderate effect (*d* ≥ 0.50); large effect (*d* ≥ 0.80).

**Table 3:** Fatty acid profile (% of total hepatic triacylglycerols) of the hepatic triacylglycerols from obese Zucker rats treated or not with pterostilbene (30 mg/kg body weight/day for six weeks).

Fatty Acid	Control	PT		Effect Size ^a^
**C14:0**	1.58 ± 0.27	1.67 ± 0.04	NS	
**C16:0**	35.02 ± 1.44	33.56 ± 0.87	NS	
**C16:1 *cis*-9 *n*-7**	10.56 ± 0.68	9.45 ± 0.49	NS	
**C18:0**	1.99 ± 0.17	2.41 ± 0.28	NS	
**C18:1 *cis*-9 *n*-9**	22.82 ± 1.86	22.11 ± 1.27	NS	
**C18:1 *cis*-11 *n*-7**	7.63 ± 1.40	6.89 ± 0.79	NS	
**C18:2 *n*-6**	14.59 ± 1.36	18.85 ± 1.17	*p* = 0.04	1.07
**C18:3 *n*-3**	0.45 ± 0.07	0.68 ± 0.08	*p* = 0.05	0.97
**C20:0**	4.39 ± 2.14	3.21 ± 0.42	NS	
**C22:0**	0.04 ± 0.02	0.04 ± 0.02	NS	
**C20:4 *n*-6**	0.93 ± 0.18	1.14 ± 0.27	NS	
**∑SFA**	43.02 ± 1.15	40.89 ± 0.82	NS	
**∑MUFA**	41.00 ± 1.59	38.44 ± 1.13	NS	
**∑PUFA**	15.98 ± 1.50	20.67 ± 1.36	*p* = 0.04	1.04

Pterostilbene (PT). ^a^ Cohen’s recommendation for the interpretation of effect size: Little effect (*d* ≥ 0.20); moderate effect (*d* ≥ 0.50); large effect (*d* ≥ 0.80).

**Table 4:** Fatty acid profile (% of total hepatic phospholipids) of the hepatic phospholipids from obese Zucker rats treated or not with pterostilbene (30 mg/kg body weight/day for six weeks).

Fatty Acid	Control	PT		Effect Size ^a^
**C14:0**	0.77 ± 0.12	0.50 ± 0.04	NS	
**C16:0**	23.34 ± 0.49	21.42 ± 0.25	NS	
**C16:1 *cis*-9 *n*-7**	3.64 ± 0.65	2.13 ± 0.25	*p* = 0.06	−0.98
**C18:0**	19.11 ± 1.13	20.93 ± 0.77	NS	
**C18:1 *cis*-9 *n*-9**	7.06 ± 1.16	4.65 ± 0.37	NS	
**C18:1 *cis*-11 *n*-7**	4.00 ± 0.45	2.83 ± 0.16	*p* = 0.04	−1.11
**C18:2 *n*-6**	7.06 ± 0.29	8.20 ± 1.03	NS	
**C18:3 *n*-3**	0.13 ± 0.04	0.11 ± 0.04	NS	
**C20:4 *n*-6**	28.04 ± 1.82	30.89 ± 0.78	NS	
**C20:5 *n*-3**	0.03 ± 0.01	0.03 ± 0.01	NS	
**C22:6 *n*-3**	6.81 ± 0.57	8.31 ± 0.35	*p* = 0.05	1.01
**∑SFA**	43.22 ± 0.14	42.85 ± 0.54	NS	
**∑MUFA**	14.71 ± 2.25	9.61 ± 0.74	*p* = 0.06	−0.97
**∑PUFA**	42.07 ± 2.23	47.53 ± 0.91	*p* = 0.05	1.03

Pterostilbene (PT). ^a^ Cohen’s recommendation for the interpretation of effect size: Little effect (*d* ≥ 0.20); moderate effect (*d* ≥ 0.50); large effect (*d* ≥ 0.80).
